# The bacterial type III-secreted protein AvrRps4 is a bipartite effector

**DOI:** 10.1371/journal.ppat.1006984

**Published:** 2018-03-30

**Authors:** Morgan K. Halane, Sang Hee Kim, Benjamin J. Spears, Christopher M. Garner, Conner J. Rogan, Elizabeth C. Okafor, Jianbin Su, Saikat Bhattacharjee, Walter Gassmann

**Affiliations:** 1 Division of Plant Sciences, University of Missouri, Columbia, MO, United States of America; 2 Christopher S. Bond Life Sciences Center and Interdisciplinary Plant Group, University of Missouri, Columbia, MO, United States of America; 3 Division of Applied Life Science (BK 21 Plus Program), Plant Molecular Biology and Biotechnology Research Center, Gyeongsang National University, Jinju, Republic of Korea; 4 Division of Life Science, Gyeongsang National University, Jinju, Republic of Korea; 5 Division of Biological Sciences, University of Missouri, Columbia, MO, United States of America; 6 Regional Centre for Biotechnology, NCR Biotech Science Cluster, Faridabad, India; The Ohio State University, UNITED STATES

## Abstract

Bacterial effector proteins secreted into host plant cells manipulate those cells to the benefit of the pathogen, but effector-triggered immunity (ETI) occurs when effectors are recognized by host resistance proteins. The RPS4/RRS1 pair recognizes the *Pseudomonas syringae* pv. pisi effector AvrRps4. AvrRps4 is processed *in planta* into AvrRps4^N^ (133 amino acids), homologous to the N-termini of other effectors including the native *P*. *syringae* pv. tomato strain DC3000 effector HopK1, and AvrRps4^C^ (88 amino acids). Previous data suggested that AvrRps4^C^ alone is necessary and sufficient for resistance when overexpressed in heterologous systems. We show that delivering AvrRps4^C^ from DC3000, but not from a DC3000 *hopK1*^*-*^ strain, triggers resistance in the Arabidopsis accession Col-0. Delivering AvrRps4^C^ in tandem with AvrRps4^N^, or as a chimera with HopK1^N^, fully complements AvrRps4-triggered immunity. AvrRps4^N^ in the absence of AvrRps4^C^ enhances virulence in Col-0. In addition, AvrRps4^N^ triggers a hypersensitive response in lettuce that is attenuated by coexpression of AvrRps4^C^, further supporting the role of AvrRps4^N^ as a bona fide effector domain. Based on these results we propose that evolutionarily, fusion of AvrRps4^C^ to AvrRps4^N^ may have counteracted recognition of AvrRps4^N^, and that the plant *RPS4/RRS1* resistance gene pair was selected as a countermeasure. We conclude that AvrRps4 represents an unusual chimeric effector, with recognition in Arabidopsis by RPS4/RRS1 requiring the presence of both processed effector moieties.

## Author summary

An important component of the plant immune system relies on the detection of pathogen-derived effectors by immune receptors called resistance proteins. Bacterial pathogens inject effectors into the host cell via a dedicated secretion system to suppress defenses and to manipulate the physiology of the host cell to the pathogen's advantage. Usually, a single resistance protein recognizes a single effector, but an increasing number of exceptions and elaborations on this one-to-one relationship are known. The plant Arabidopsis uses a pair of resistance proteins, RRS1 and RPS4, to detect the effector AvrRps4. After injection into the cell, AvrRps4 is cleaved into two protein parts, and it had been assumed that only the C-terminal part needs to be present to trigger RPS4/RRS1. We show here that both AvrRps4 parts are required for triggering resistance in Arabidopsis, and that the N-terminal part, which previously had been assumed to only function in effector secretion into the host cell, in fact on its own has some functions of an effector. This conclusion is supported by the observation that the N-terminal part of AvrRps4 is sufficient to trigger resistance in lettuce. The fusion of the two AvrRps4 parts may have arisen to counteract plant defenses.

## Introduction

Plants deploy a multilayered immune system to defend against invading pathogens [1–3]. The first layer of defense, pathogen-associated molecular pattern-triggered immunity (PAMP-triggered immunity), relies on membrane-localized pattern recognition receptors which recognize conserved non-self molecules from pathogens [4,5]. Detection of these molecules rapidly activates signaling cascades culminating in disease resistance. Effector molecules are secreted from pathogens into the host cell, often blocking PAMP-triggered immune responses or reprograming host cell transcription and physiology, leading to successful colonization of the host plant by the pathogen [6,7]. To counteract this, plants have evolved resistance proteins which can either directly or indirectly detect the presence of these effectors and then ramp up a robust immune response called effector-triggered immunity (ETI) [8].

The effector AvrRps4, originally identified in *Pseudomonas syringae* pv. pisi [9], has currently uncharacterized biochemical activity in host cells. Full-length AvrRps4 is 221 amino acids in length and is processed within host cells between Gly133 and Gly134 [10]. The processed N-terminal AvrRps4 fragment (AvrRps4^N^, amino acids 1–133) shares 75% identity with the N-terminus of HopK1, a native effector of the *Pseudomonas syringae* pv. tomato strain DC3000 (DC3000) that is predominantly used for bacterial delivery of effectors in Arabidopsis studies. HopK1 is a 338 amino acid protein that contributes significantly to the virulence of DC3000. Similarly to AvrRps4, HopK1 is processed in plant cells between amino acids Gly133 and Gly134 [11]. AvrRps4^C^ (amino acids 134–221) is a coiled-coil protein which interacts with WRKY proteins, a plant-specific class of transcription factors of which several have been implicated in defense against pathogens [12–14]. The resistance protein RESISTANCE TO RALSTONIA SOLANACEARUM1 (RRS1) contains an integrated WRKY domain at the C-terminus [15]. RRS1 works as a pair with another resistance protein, RESISTANCE TO PSEUDOMONAS SYRINGAE4 (RPS4), to trigger defense against pathogens secreting AvrRps4 [16,17].

RRS1 and RPS4 belong to the Toll/Interleukin-1 Receptor—Nucleotide-Binding—Leucine-Rich Repeat (TNL) class of resistance proteins [15,18] and, apart from AvrRps4, also detect the *Ralstonia solanacearum* effector PopP2 and an unknown *Colletotrichum higginsianum* effector [16,17]. As a paradigm for the integrated decoy model [19] and TNL signaling, it is of paramount interest to establish how RPS4/RRS1 are activated by the presence of these unrelated effectors. PopP2 has been shown to acetylate host proteins, including RRS1 in its WRKY domain at amino acid positions that mediate contact with DNA. These findings led to the model that PopP2 evolved to target host WRKYs important in resistance but are baited by the decoy WRKY in RRS1, activating RPS4 and subsequent defense [12,13,20]. Previously it was reported that AvrRps4^C^ was sufficient to trigger a hypersensitive response (HR) in turnip [10], and that AvrRps4^C^ interacts, directly or indirectly, with the WRKY domain of RRS1 as well [13].

Additional components of RPS4/RRS1 activation by AvrRps4 are its interaction in the cytoplasm and nucleus with ENHANCED DISEASE SUSCEPTIBILITY1 (EDS1), a positive regulator of both basal resistance and ETI, and the interaction between EDS1 and RPS4 [21,22]. EDS1 is required for ETI mediated by the TNL class of resistance proteins [23,24]. This may suggest that RPS4/RRS1 guard EDS1, but the connection between the two models of AvrRps4 recognition is not understood. It was recently confirmed that AvrRps4 does indeed interact with EDS1 even though it was controversial in previous reports [25], but gaps in knowledge for how this interaction connects to RPS4/RRS1 activation remain. One complication in determining a mechanism is the fact that these resistance protein complexes are dynamic and sensitive to shifts in protein amounts. A careful study demonstrated that the composition and localization of RPS4/RRS1 complexes in transient expression studies vary with the absence or presence of other expressed proteins, specifically EDS1 and PAD4 [25]. The complexity of the system becomes evident when considering that this study did not include other well-known protein partners such as the co-chaperone SGT1 and the immune adaptor protein SRFR1, and like previous studies from several groups relied on static protein overexpression. Specifically, SRFR1 does not localize to the soluble cytoplasmic fraction in *N*. *benthamiana*, or in Arabidopsis when expressed from its native promoter [21,26]. This may be important in localizing RPS4 [27] and a sub-pool of EDS1 [21] to the microsomal cytoplasmic fraction, where disruption of protein interactions by AvrRps4 was reported [21].

Here we show that AvrRps4^N^ directly interacts with EDS1, suggesting that AvrRps4^N^ possesses effector functions outside of it being a signal peptide for secretion and a transit peptide for chloroplast localization. In addition, we endeavored to determine the molecular basis of AvrRps4 triggering RPS4/RRS1 by introducing bacterially delivered AvrRps4 to ascertain function of separate domains of this effector. We provide evidence that HopK1^N^ and AvrRps4^N^ at natural expression levels redundantly contribute to RPS4/RRS1-mediated immunity in the presence of AvrRps4^C^. Consequently, both AvrRps4^C^ and either HopK1^N^ or AvrRps4^N^ are required for the full ETI-inducing activity of AvrRps4 in Arabidopsis. Additionally, AvrRps4^N^ increases bacterial virulence when overexpressed in Col-0, and triggers a robust HR when expressed in cultivars of *Lactuca sativa* (lettuce) while AvrRps4^C^ triggers no such response. We propose that separate interactions of AvrRps4^N^ with EDS1 and of AvrRps4^C^ with RRS1 are components of RPS4/RRS1 activation.

## Results

### AvrRps4^N^ interacts with EDS1

**Fig 1:**
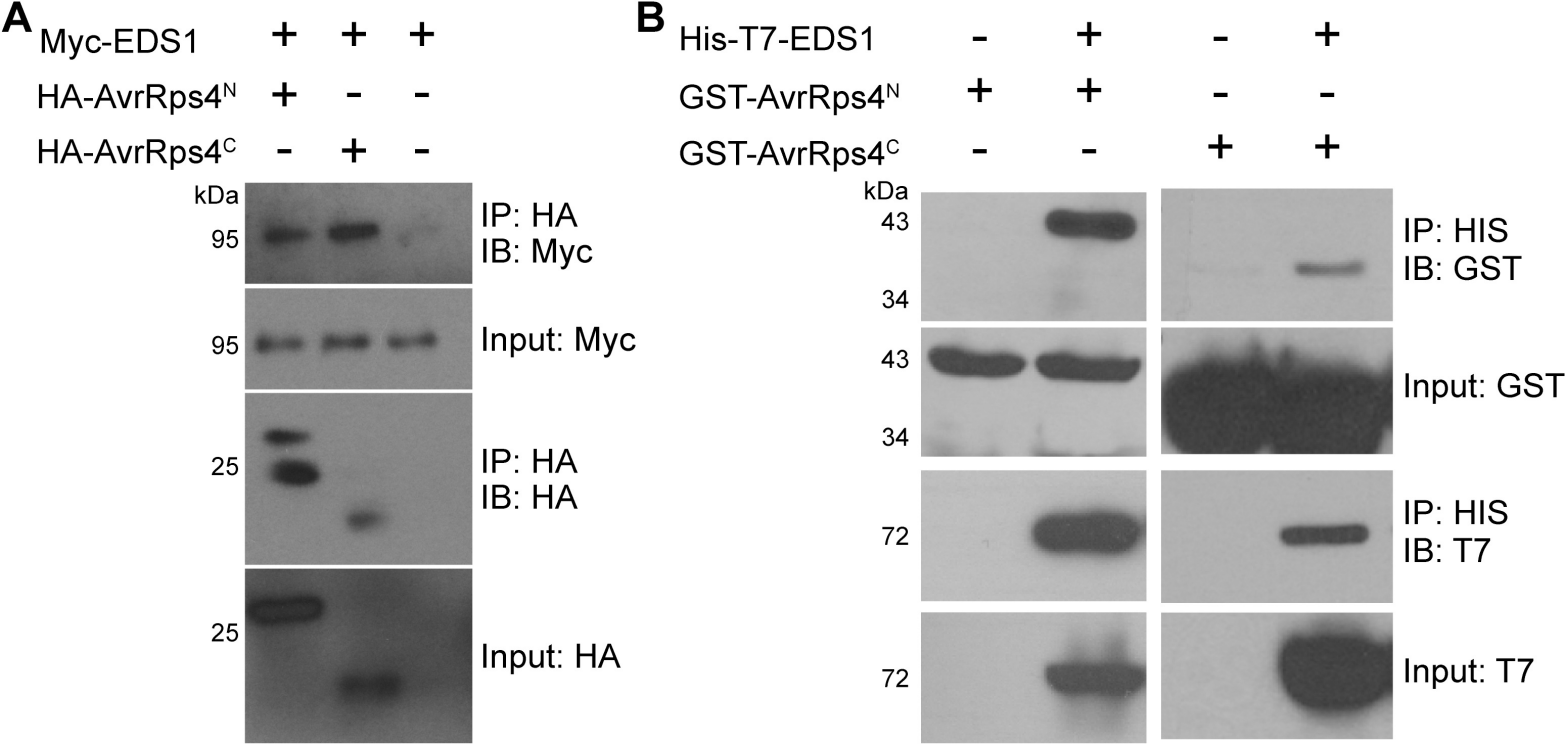
AvrRps4^N^ and AvrRps4^C^ directly interact with EDS1. (A) Co-IP of microsomal EDS1 with AvrRps4^N^ and AvrRps4^C^ expressed in *N*. *benthamiana*. This experiment was repeated once with similar results. (B) *In vitro* interaction of AvrRps4^N^ and AvrRps4^C^ with EDS1 in *E*. *coli*. Proteins were pulled down and subjected to immunoblot analysis with either His or T7 antibodies. This experiment was repeated once with similar results.

We previously showed that N-terminally tagged full-length AvrRps4 interacts with EDS1 *in vivo* and *in vitro*, indicating that the interaction is direct and possibly mediated by AvrRps4^N^ [21]. To specifically investigate which AvrRps4 fragment binds to EDS1 we repeated these experiments with N-terminally tagged AvrRps4^N^. Consistent with previous biochemical fractionations of HA-AvrRps4-expressing cells [21], apart from a nuclear pool AvrRps4^N^ transiently expressed in *Nicotiana benthamiana* was detected in the cytoplasmic microsomal fraction. *In vivo* co-immunoprecipitation of AvrRps4^N^ and EDS1 in the microsomal fraction confirmed that AvrRps4^N^ is sufficient for this interaction (Fig 1A, S1A Fig). Association of AvrRps4^C^ and EDS1 was also observed. Additionally, AvrRps4^N^ and AvrRps4^C^ specifically associated with EDS1 *in vitro* (Fig 1B, S1B Fig). Based on the relative ratios of input and pulldown protein amounts, the interaction of EDS1 with AvrRps4^N^ appeared to be stronger than with AvrRps4^C^. This interaction implies a previously uncharacterized function of AvrRps4^N^ in bacterial virulence or avirulence in addition to type III secretion and chloroplast localization [11], since effectors commonly target key hubs of resistance [28].

### DC3000-secreted full-length AvrRps4 delivered into host cells with the AvrRpm1 signal peptide triggers resistance

To identify possible roles of AvrRps4^N^ in virulence or triggering resistance at natural expression levels we utilized the vector pVSP_*Ps*SPdes [29], which enables secretion from *Pseudomonas syringae* into host cells of parts of cloned effectors such as AvrRps4^C^ (S2 Fig). This vector includes an AvrRpm1 signal peptide for protein secretion and an HA epitope tag for protein detection. Three additional constructs, each with the AvrRpm1 signal peptide removed, were also constructed. All constructs were driven by the AvrRpm1 promoter. As expected, full-length AvrRps4 with or without the AvrRpm1 signal peptide triggered resistance to DC3000 (S3 Fig). AvrRps4^N^ and AvrRps4^C^ with or without the AvrRpm1 signal peptide failed to restore the resistance phenotype of full-length AvrRps4. AvrRps4^C^ without the AvrRpm1 signal peptide is not secreted from DC3000 and served as a negative control. In contrast, the negative result with AvrRpm1SP-AvrRps4^C^ superficially did not support the hypothesis based on turnip HR that AvrRps4^C^ is sufficient for resistance [10].

### The AvrRpm1 signal peptide mislocalizes AvrRps4 proteins to host membranes

**Fig 2:**
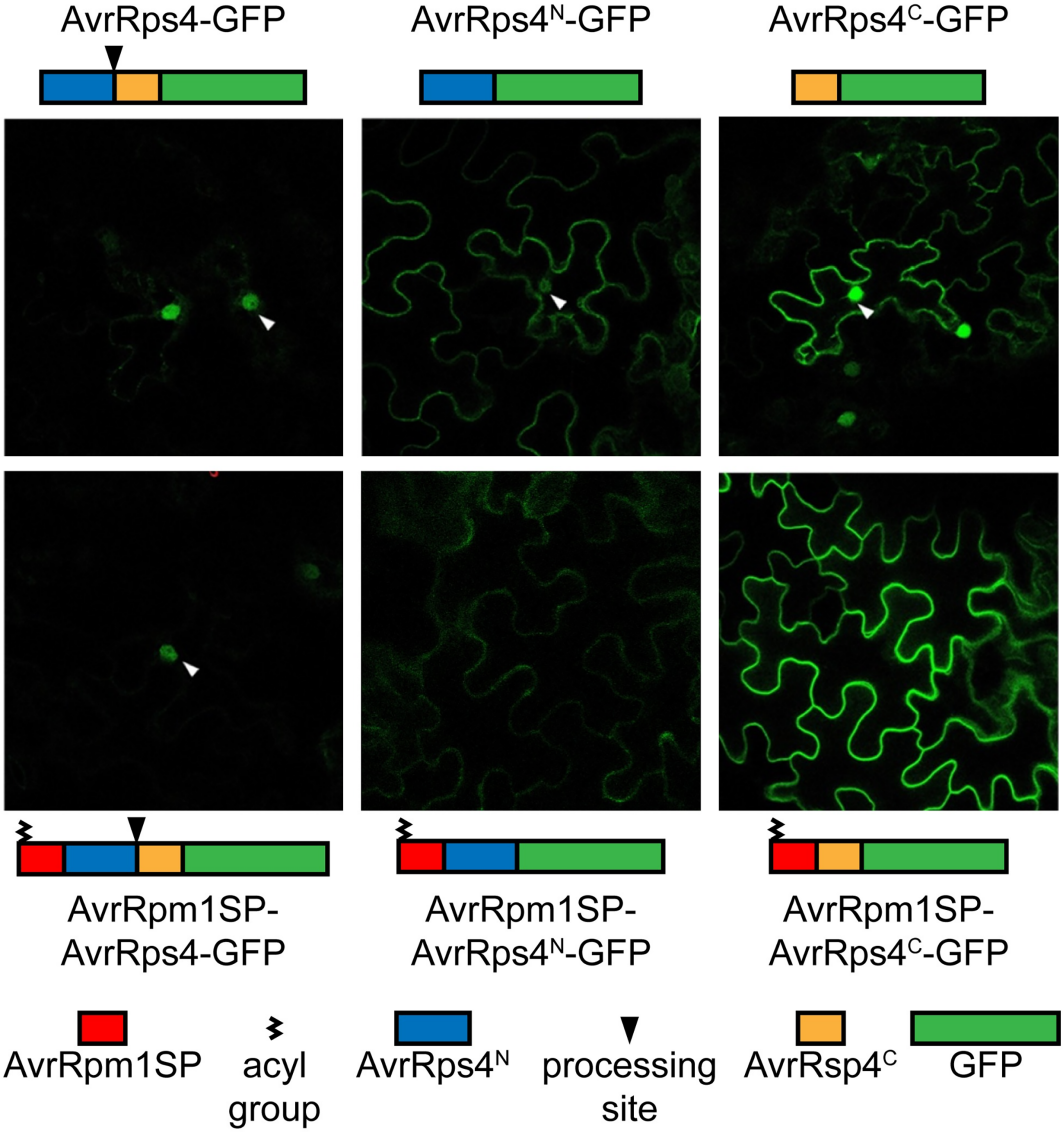
Localization of GFP-tagged AvrRps4 variants. AvrRps4 full-length, AvrRps4^N^, and AvrRps4^C^ with or without the AvrRpm1 signal peptide were C-terminally tagged with GFP and expressed in *N*. *benthamiana*. Cells were visualized after two days. For each construct, quantification of twenty random cells showed no variation in subcellular localization of GFP signal. Arrows indicate nuclei. This experiment was repeated twice with similar results.

To further investigate why AvrRps4^C^ delivered into cells via the AvrRpm1 signal peptide did not trigger resistance we explored the possibility of protein mislocalization. Previously, it was shown that AvrRpm1 is acylated at its second amino acid residue, Gly, leading to AvrRpm1 localization to the host cell plasma membrane [30]. Host membrane tethering of the protein was disrupted by mutating Gly to Ala. To test for mislocalization of our AvrRps4 proteins we made C-terminal translational GFP fusions of full-length AvrRps4, AvrRps4^N^, and AvrRps4^C^ with or without the AvrRpm1 signal peptide. AvrRps4 transiently expressed in *N*. *benthamiana* was shown not to be imported into chloroplasts [11], and AvrRps^N^-GFP and AvrRps4^C^-GFP localized to the nucleus in addition to the cytoplasm (Fig 2). Therefore, localization of GFP signal within the nucleus and at the plasma membrane can be used as an assay for extrachloroplastic processing of full-length AvrRps4 and mislocalization of AvrRpm1SP-AvrRps4^N/C^ proteins, respectively. Indeed, the AvrRpm1 signal peptide disrupted the localization of AvrRps4 when transiently expressed in *N*. *benthamiana* cells (Fig 2). Whereas nuclear signals were observed with full-length AvrRps4-GFP, AvrRps4^N^-GFP, and AvrRps4^C^-GFP, these signals were abolished in the AvrRpm1 signal peptide fusions of AvrRps4^N^ and AvrRps4^C^. However, a nuclear signal was still detected in AvrRpm1 signal peptide fusion with full-length AvrRps4, indicating that processed AvrRps4^C^-GFP was liberated and gained entry into the nucleus. Coexpression of GFP-tagged AvrRps4 proteins with free RFP to mark the cytoplasm and nucleus confirmed the presence (AvrRps4-GFP, AvrRps4^N^-GFP, AvrRps4^C^-GFP, and AvrRpm1SP-AvrRps4-GFP) and absence (AvrRpm1SP-AvrRps4^N^ and AvrRpm1SP-AvrRps4^C^) of nuclear signal (S4 Fig). Assays with delivery by DC3000 confirmed that AvrRpm1SP-AvrRps4 is still processed in Arabidopsis cells (see below).

### HopK1^N^ is functionally redundant to AvrRps4^N^ in effector-triggered immunity

**Fig 3:**
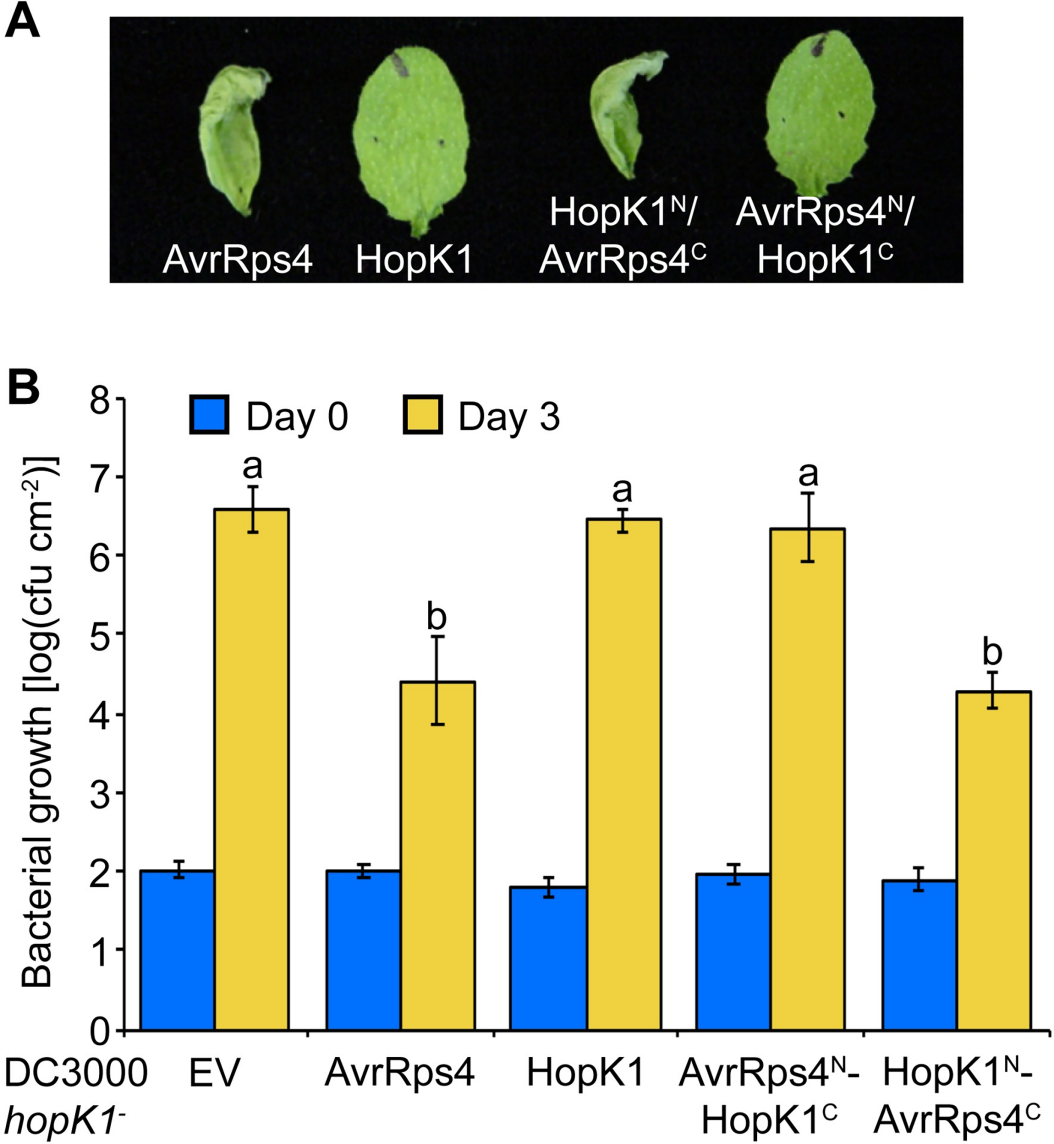
HopK1^N^/AvrRps4^C^ chimeras are functional in ETI. (A) The indicated AvrRps4 and HopK1 constructs were delivered from the *P*. *fluorescens* strain EtHAn into Arabidopsis Ws-0. HR response was observed after 24 hours. This experiment was repeated at least twice with similar results. (B) *In planta* bacterial growth analysis of DC3000 *hopK1*^*-*^ secreting indicated wild-type or chimeric effectors. Ws-0 plants were inoculated with 5x10^4^ cfu/mL suspensions of bacteria. Values are averages from two independent experiments with triplicate samples, and error bars denote standard deviation, with letters indicating statistically significant differences (*P*<0.0001).

AvrRps4^N^ is 75% identical to HopK1^N^, the N-terminus of the native DC3000 effector HopK1. Both AvrRps4 and HopK1 are processed at the same amino acid residue [11]. This remarkable conservation led us to test whether HopK1^N^ and AvrRps4^N^ could redundantly trigger immunity in the presence of AvrRps4^C^. Chimeras of HopK1^N^-AvrRps4^C^ and AvrRps4^N^-HopK1^C^ were constructed. For this series of experiments, we used the Arabidopsis accession Wassilewskija (Ws-0), which like Col-0 possesses functional *RPS4* and *RRS1* for AvrRps4 recognition [9,16,17]. In contrast to Col-0, which displays resistance to AvrRps4 uncoupled from HR, Ws-0 responds to AvrRps4 with a strong HR, thus enabling the use of non-pathogenic strains for HR assays in the absence of additional effectors [18,31]. When delivered via the *Pseudomonas fluorescens* Effector-to-Host Analyzer system (Pfo EtHAn) [32] the AvrRps4 full-length protein triggered a strong HR in Ws-0 after 24 hours (Fig 3A). HopK1 delivered from Pfo EtHAn did not trigger an HR. Interestingly, a HopK1^N^-AvrRps4^C^ chimera indeed caused a strong HR, whereas an AvrRps4^N^-HopK1^C^ chimera did not. To confirm these results, *in planta* bacterial growth assays were performed using chimeras delivered from a DC3000 *hopK1*^-^ mutant into Ws-0. The HopK1^N^-AvrRps4^C^ chimera triggered resistance similar to full-length AvrRps4, whereas AvrRps4^N^-HopK1^C^ supported bacterial growth similar to empty vector and HopK1 (Fig 3B). Additionally, resistance in Ws-0 to chimeric HopK1^N^-AvrRps4^C^ required wild-type copies of *EDS1* and *RPS4* to the same extent as resistance to AvrRps4 (S5 Fig). Taken together, these results indicate that AvrRps4^N^ and HopK1^N^ function redundantly in triggering ETI in the presence of AvrRps4^C^ in the Arabidopsis system.

### Full-length AvrRps4 fused to the AvrRpm1 signal peptide does not trigger immunity in the absence of HopK1

**Fig 4:**
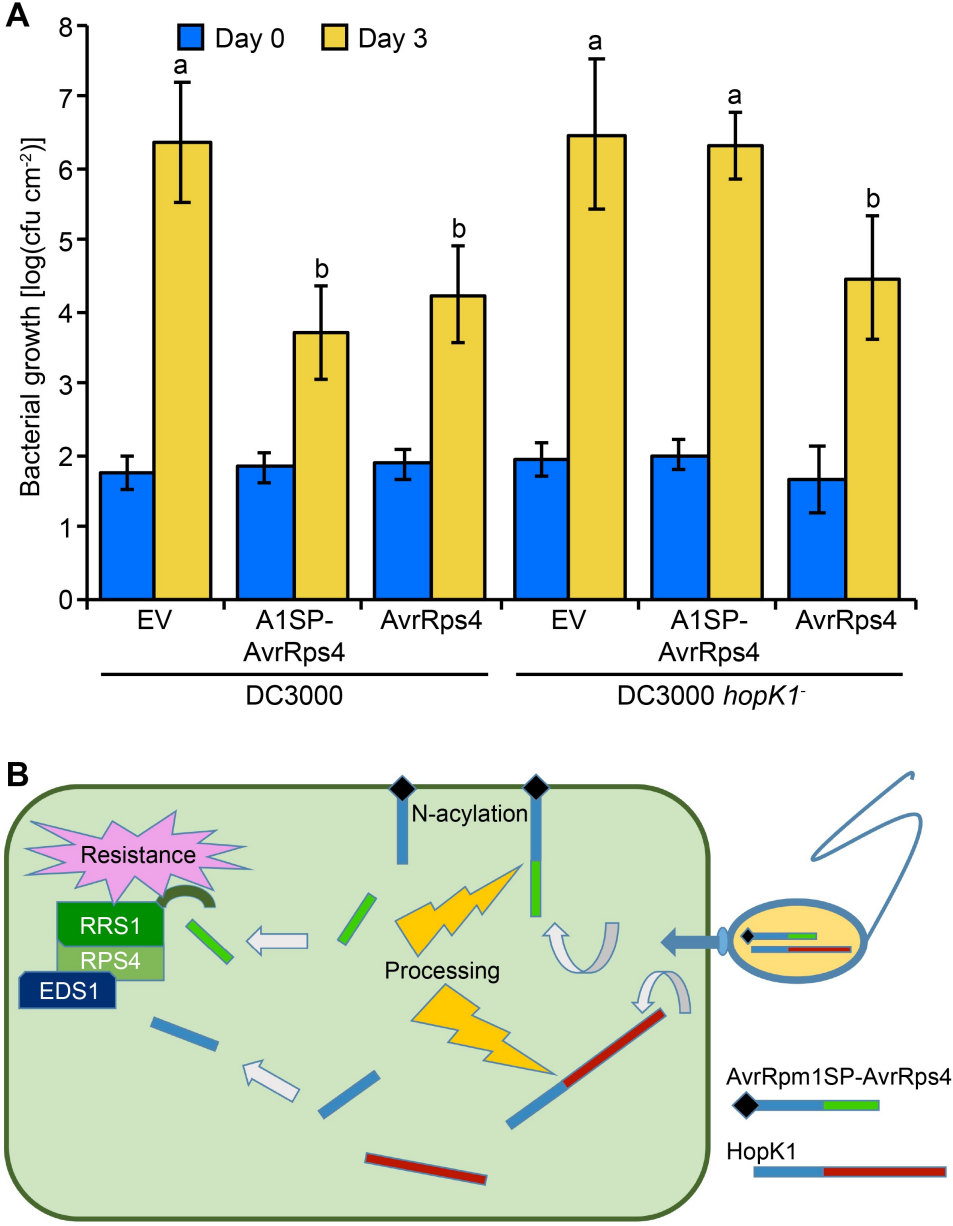
ETI is attenuated when AvrRpm1SP-AvrRps4 is delivered from DC3000 *hopK1*^-^ compared to DC3000. (A) Col-0 plants were inoculated with 5x10^4^ cfu/mL suspensions of either wild-type DC3000 or the *hopK1* deletion strain DC3000 *hopK1*^*-*^ containing empty vector (EV), or expressing AvrRpm1SP-AvrRps4 (A1SP-AvrRps4) or AvrRps4. Values are averages from three (DC3000) or four (DC3000 *hopK1*^*-*^) independent experiments with triplicate samples, and error bars denote standard deviation, with letters indicating statistically significant differences (*P*<0.0001). (B) Model of HopK1^N^ contribution to AvrRpm1SP-AvrRps4-triggered immunity.

Since HopK1 is an effector native to DC3000, we hypothesized that processed HopK1^N^ may substitute for mislocalized AvrRpm1SP-AvrRps4^N^ delivered from DC3000 in bacterial growth assays. To address this question, we introduced all of our AvrRps4 constructs into DC3000 *hopK1*^-^ [11]. Surprisingly, whereas the AvrRpm1SP-AvrRps4 full-length protein delivered from DC3000 into Arabidopsis Col-0 triggered resistance similar to wild-type AvrRps4, the same construct delivered from DC3000 *hopK1*^-^ failed to reconstitute this strong resistance phenotype (Fig 4A). Protein secretion assays confirmed that AvrRps4 proteins with or without AvrRpm1SP were still being secreted from DC3000 and DC3000 *hopK1*^-^ (S6 Fig). After harvesting tissue from Col-0 leaves infiltrated with AvrRpm1SP-AvrRps4 from DC3000 *hopK1*^-^ we could detect both the full-length and processed forms of this fusion protein (S7 Fig), consistent with results obtained by transient expression in *N*. *benthamiana* (Fig 2). Together with our localization data, this shows that although AvrRpm1SP-AvrRps4^N^ mislocalizes to the host membrane, AvrRps4^C^ is still liberated after processing when full-length AvrRps4 is delivered into host cells via the AvrRpm1 signal peptide. Consequently, AvrRpm1SP-AvrRps4 should limit DC3000 *hopK1*^-^ multiplication in Col-0 leaves if AvrRps4^C^ were sufficient for triggering resistance. In individual replicate experiments AvrRpm1SP-AvrRps4 yielded partial resistance when compared to our empty vector controls, even though in aggregate this difference was not statistically significant (Fig 4A). This suggests that AvrRps4^C^ alone may trigger a partial resistance response, or that the AvrRps4^N^ pool is not completely mislocalized under certain conditions. Taken together, these results show that the addition of HopK1^N^ or AvrRps4^N^ in conjunction with AvrRps4^C^ enhances any resistance triggered by AvrRps4^C^ alone (see model Fig 4B).

### Bacteria secreting AvrRps4^N^ in tandem with AvrRpm1SP-AvrRps4 trigger resistance comparable to levels with wild-type AvrRps4

**Fig 5:**
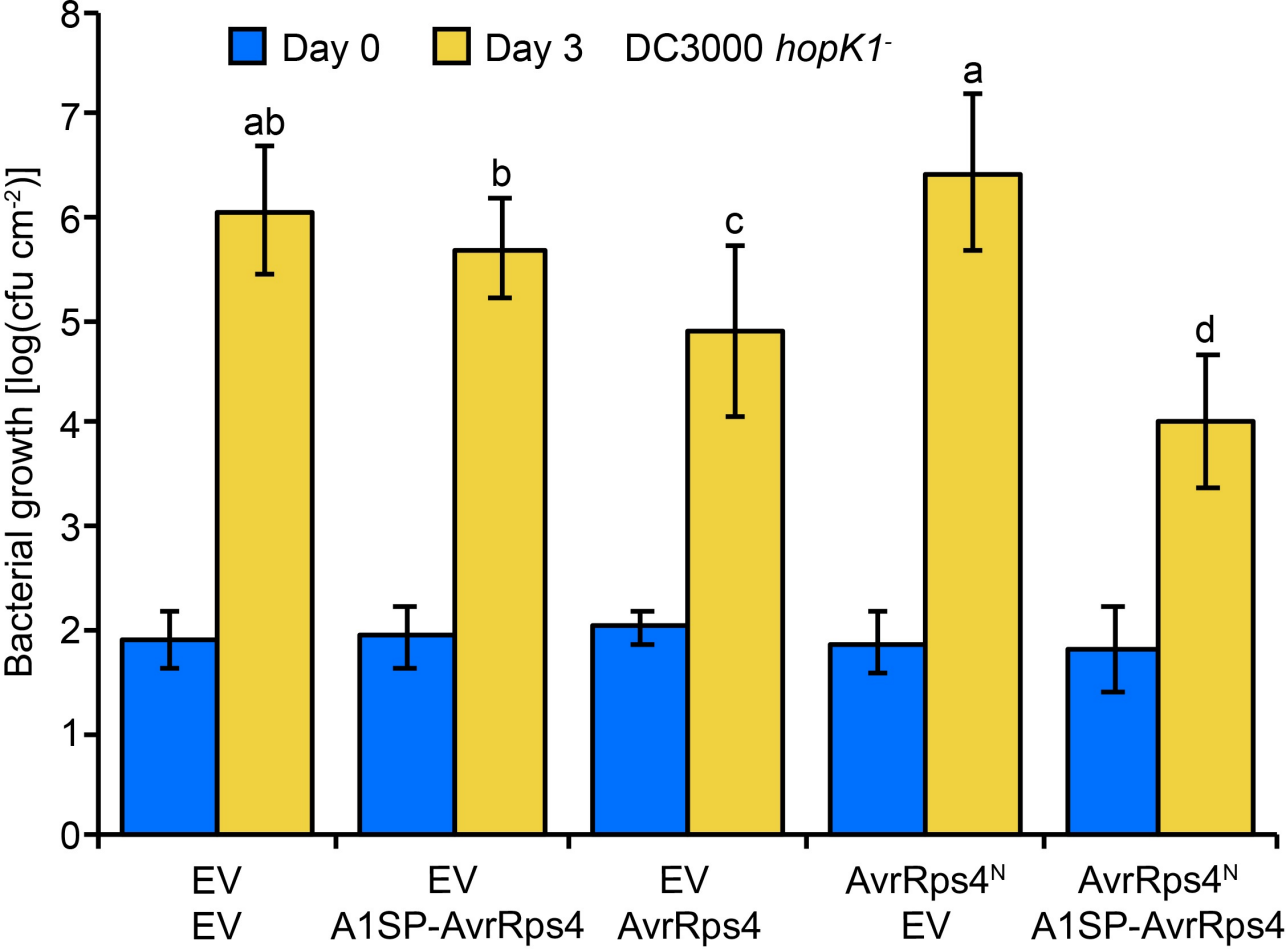
Bacteria secreting AvrRps4^N^ and AvrRps4^C^ in tandem trigger resistance comparable to wild-type AvrRps4. Col-0 plants were inoculated with 5x10^4^ cfu/mL suspensions of the indicated DC3000 *hopK1*^*-*^ strains. AvrRps4 variants were delivered from separate broad host range plasmids (top: pML123 constructs; bottom: pVSP61 constructs). Error bars denote standard deviation. Values are averages from four independent experiments with triplicate samples, and error bars denote standard deviation, with letters indicating statistically significant differences (*P*<0.05).

To test whether this enhanced resistance could be recapitulated in the bacterial system we designed a dual-vector system where DC3000 *hopK1*^-^ mutants were made to secrete effectors from two different broad host range plasmids, pVSP61 or pML123, thus ensuring that any cell in contact with this strain would receive both proteins via bacterial delivery. When AvrRps4^N^ was delivered from pML123 in tandem with AvrRpm1SP-AvrRps4 from pVSP61 in DC3000 *hopK1*^-^, the resistance phenotype was similar if not stronger to that of full-length AvrRps4 (Fig 5). These findings further confirm that AvrRps4^N^ and AvrRps4^C^ together function in triggering a full ETI response.

### AvrRps4^N^ alone functions as a virulence factor in Arabidopsis

**Fig 6:**
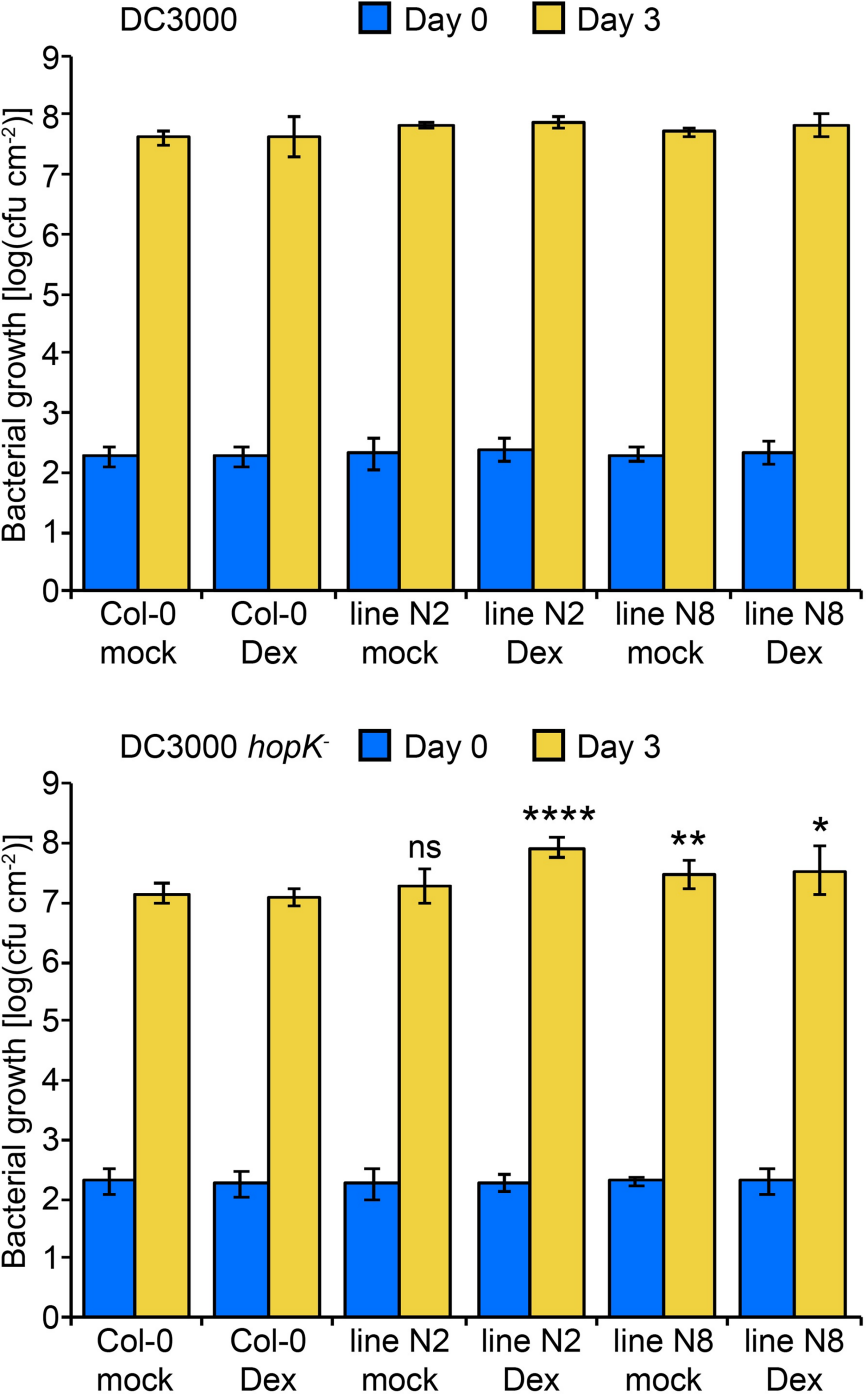
AvrRps4^N^ overexpression enhances bacterial virulence. *In planta* bacterial growth was measured in two independent Col-0 AvrRps4^N^ lines (N2 and N8) and untransformed Col-0 after inoculation with DC3000 (top) and DC3000 *hopK1*^*-*^ (bottom) at 5x10^4^ cfu/mL in the presence of dexamethasone (Dex) or ethanol (mock). Values are averages from two independent experiments with quadruplicate samples, and error bars denote standard deviation. Asterisks denote statistically significant differences compared to bacterial growth in mock- and Dex-treated Col-0 based on two-tailed Student's t-tests (ns: non-significant, *P<0.05, **P<0.01, ****P<0.0001).

AvrRps4^N^ interacted with EDS1 and was required for AvrRps4-triggered immunity. Additionally, in some individual experiments bacterial growth was enhanced by AvrRps4^N^, even though the difference was not statistically significant in aggregate (Fig 5). We therefore asked whether AvrRps4^N^ in the absence of AvrRps4^C^ has a detectable virulence activity by generating transgenic Arabidopsis lines overexpressing AvrRps4^N^ from a Dex-inducible promoter. Bacterial growth of DC3000 *hopK1*^-^ was approximately ten times higher in Dex-treated line 2 (N2), and five times higher in mock- or Dex-treated line 8 (N8) than in mock- or Dex-treated Col-0, whereas the growth of wild-type DC3000 was similar in all plants tested (Fig 6). This indicates that the virulence function of AvrRps4^N^ in DC3000 is redundant to that of native HopK1^N^. AvrRps4^N^ protein and transcripts were increased in both N2 and N8 within 24 hours after Dex treatment (S8 Fig). Consistent with the bacterial growth curve assays, *avrRps4*^*N*^ expression was leakier in N8 than in N2, and expression of the defense marker gene *PR1* was inversely correlated with *avrRps4*^*N*^ mRNA levels (S8A Fig). These results strongly suggest that AvrRps4^N^ contributes to bacterial virulence in DC3000 lacking HopK1, further supporting that AvrRps4 is a bipartite effector.

### AvrRps4^N^ expression in lettuce triggers a hypersensitive response which is attenuated in the presence of AvrRps4^C^

**Fig 7:**
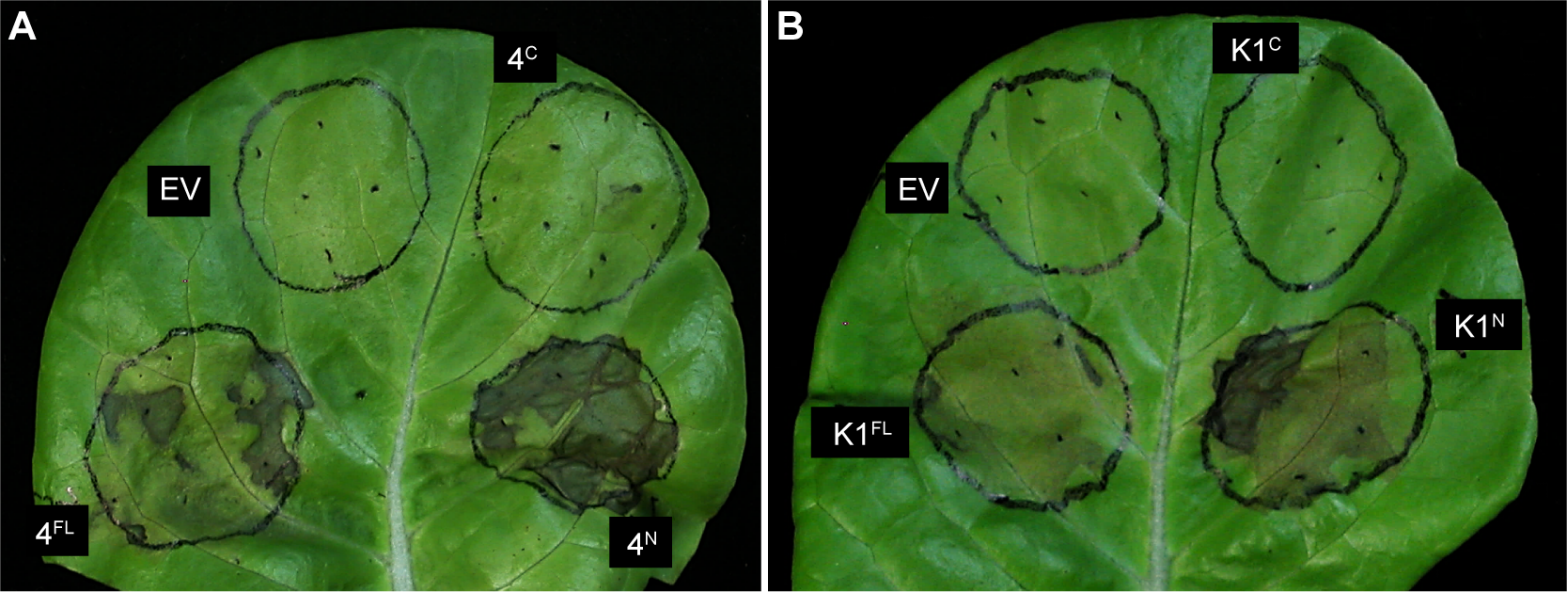
AvrRps4^N^ and HopK1^N^ are sufficient to trigger a hypersensitive response in lettuce. (A) Agrobacterium C58C1 containing the constructs for transient expression of N-terminally HA-tagged AvrRps4 full-length (4^FL^), AvrRps4^N^ (4^N^), AvrRps4^C^ (4^C^), and HA-pBA empty vector (EV) was infiltrated into *Lactuca sativa* cv. Kordaat at an O.D. of 0.3. (B) Equivalent experiment with HopK1 full-length (K1^FL^), HopK1^N^ (K1^N^), HopK1^C^ (K1^C^), and HA-pBA empty vector (EV). Cell death phenotypes in (A) and (B) were visualized three days post-infiltration. These experiments were repeated four times with identical results.

**Fig 8:**
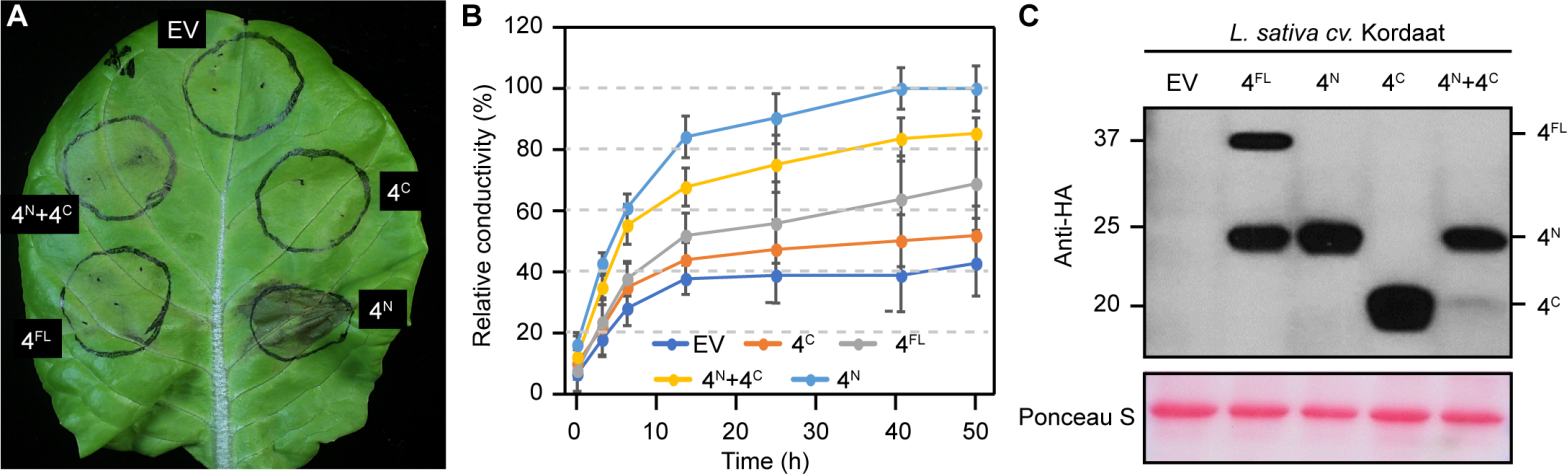
AvrRps4^N^ triggers a hypersensitive response in lettuce which is attenuated by expression of AvrRps4^C^. (A) N-terminally HA-tagged AvrRps4 full-length (4^FL^), AvrRps4^N^ (4^N^), AvrRps4^C^ (4^C^), combined AvrRps4^N^ and AvrRps4^C^ (4^N^+4^C^), or empty-vector HA-pBA (EV) were expressed in *L*. *sativa* cv. Kordaat at an O.D. of 0.3. Cell death was imaged three days after infiltration. This experiment was repeated four times with identical results. (B) Conductivity as a measure of electrolyte release by cells undergoing HR. Measurements were taken at the indicated time points after vacuum infiltration of lettuce leaf discs with ddH_2_O, 36 hours after infiltration with agrobacterium strains. Values represent averages from two independent experiments with quintuplicate samples normalized to maximal conductivity observed with AvrRps4^N^ at 48 h, and error bars denote SD. (C) Western blots confirming expression of proteins in *L*. *sativa*.

It was previously shown that full-length AvrRps4 and HopK1 expressed in several lettuce cultivars resulted in an HR [33]. Interestingly, the pattern of the variable response between cultivars was identical between AvrRps4 and HopK1, leading us to hypothesize that the N-terminal moiety of each respective effector was sufficient for such a response. To test this, we first confirmed that AvrRps4 and HopK1 could reproducibly trigger HR on *Lactuca sativa* cv. Kordaat. We then expressed AvrRps4^N^, HopK1^N^, AvrRps4^C^, and HopK1^C^ and monitored cell death (Fig 7). Indeed, AvrRps4^N^ and HopK1^N^ triggered a strong HR whereas neither AvrRps4^C^ nor HopK1^C^ induced HR. We consistently observed that full-length AvrRps4 and full-length HopK1 induced weaker cell death phenotypes when compared to AvrRps4^N^ or HopK1^N^, suggesting the C-terminal peptides may suppress HR induced by the N-terminal peptides. Importantly, AvrRps4^C^ co-expressed with AvrRps4^N^ attenuated the HR caused by AvrRps4^N^ yielding HR phenotypes similar to those caused by AvrRps4 full-length (Fig 8A). To quantify the level of HR we performed electrolyte leakage experiments. These showed that AvrRps4^N^ induced a faster and stronger HR than any other treatment (Fig 8B). Western blot analysis confirmed that AvrRps4^N^, AvrRps4^C^ as well as full-length AvrRps4 were expressed in *L*. *sativa*, even though levels of AvrRps4^C^ were variable when co-expressed with AvrRps4^N^ (Fig 8C).

## Discussion

We have demonstrated that AvrRps4 is a bipartite protein, where AvrRps4^N^ functions in three distinct ways, 1) triggering of resistance in the presence of AvrRps4^C^ in Arabidopsis Col-0, 2) serving as a virulence factor in the absence of AvrRps4^C^ in Col-0, and 3) triggering defense responses in lettuce. These functions of AvrRps4^N^ are consistent with functions of an effector and with its interaction with the positive regulator of immunity EDS1 shown here. Sequence conservation across the entire N-terminal moieties of AvrRps4, HopK1, and the *Xanthomonas campestris* pv. *vesicatoria* effector XopO led us to hypothesize that AvrRps4^N^ has functions that extend beyond a T3SS signal and chloroplast transit peptide, which usually are encoded in the first 20–50 amino acids of a peptide [34]. Indeed, the putative effector HopAQ1 is a relatively small 83 amino acid protein from DC3000 with a 44 amino acid signal peptide highly similar to that of AvrRps4 and HopK1 and a dissimilar sequence in its uncleaved C-terminal 39 amino acids [35,36]. While bacterial effectors with more than one distinct functional domain, such as AvrPtoB [37], have been described before, AvrRps4 so far appears unique in that two effector moieties are required to trigger resistance in Arabidopsis when present at native levels.

Both AvrRps4 and HopK1 are processed in plant cells between amino acids Gly133 and Gly134. It was suggested that this processing occurs exclusively in chloroplasts and that the N-terminal 20 amino acids encode a chloroplast transit peptide [11]. HopAQ1 lacks the proposed C-terminal effector domain of AvrRps4^N^ and is predicted to translocate to chloroplasts, although such predictions may be an artifact of prediction models and the similarity of T3SS and chloroplast targeting sequences [38]. We observed processing of AvrRps4 in *N*. *benthamiana* and Arabidopsis even with N-terminal fusions such as those to AvrRpm1SP. Since such fusions are predicted to block chloroplast import we propose that a subpool of AvrRps4 is processed in the cytoplasm, and that AvrRps4 has multiple virulence targets in multiple compartments. Based on our results it appears that AvrRps4-triggered ETI is initiated in the cytoplasm or nucleus. This does not exclude virulence functions of chloroplast-localized AvrRps4 [11].

Consistent with results presented here, a recent study also failed to detect chloroplast localization of full-length or processed AvrRps4 in Arabidopsis or *N*. *benthamiana* cells [39]. Interestingly, in this study bacterial delivery of AvrRpm1SP-AvrRps4^C^ triggered measurable resistance in Col-0. To detect bacterial delivery and effector localization in the host cell, effector constructs were tagged with a 13-amino acid split fluorescent protein sequence and were delivered into transgenic plants expressing a split superfolder GFP protein that self-assembles. The strain used for effector delivery, the DC3000 variant *Pst* CUCPB5500, expresses HopK1 [40]. Interestingly, capture of tagged AvrRpm1SP-AvrRps4^C^ by the highly soluble superfolder GFP allowed diffusion of this construct into the nucleus [39]. These results are therefore consistent with our model that untethered AvrRps4^C^ in the presence of HopK1^N^ or AvrRps4^N^ triggers resistance.

Searches of NCBI’s protein sequence database using the BLAST algorithm [41] failed to identify examples of putative effectors with homology to AvrRps4^C^ fused to only a 50 amino acid signal peptide, but *Xanthomonas* XopAK is a homolog of HopK1^C^ fused to a signal peptide lacking the 94 amino acid effector domain of AvrRps4^N^ and HopK1^N^ [42]. We therefore propose a model where ancestral AvrRps4 and HopK1 proteins consisted of only AvrRps4^N^ or HopK1^N^ homologs, which presumably targeted hubs important to immunity such as EDS1. The interactions of these proteins with EDS1 may have triggered resistance on resistant plants, and successful bacteria evaded this resistance by terminal reassortment [36] with either AvrRps4^C^ or HopK1^C^ to suppress resistance. On the plant side, genes such as the *RPS4/RRS1* pair arose to recognize the addition of AvrRps4^C^. Evidence for this include our data which show that AvrRps4^N^ alone is sufficient to enhance bacterial virulence in Arabidopsis Col-0 when overexpressed whereas addition of AvrRps4^C^ again makes Col-0 fully resistant to bacteria expressing AvrRps4^N^ and AvrRps4^C^. The virulence function of AvrRps4^N^ was occasionally discernible in *in planta* bacterial growth experiments using DC3000 *hopK1*^*-*^ but was not significant overall, as was reported previously [11]. Low levels of bacterial AvrRps4^N^ delivery and functional redundancy, as observed with several groups of sequence-unrelated DC3000 effectors [40], likely precluded robust detection of AvrRps4^N^ virulence effects at natural protein levels.

A prediction of this model is that the capacity to recognize AvrRps4^N^/HopK1^N^ still exists in some plants. Indeed, we identified lettuce as one plant species which retains the ability to recognize AvrRps4^N^. Future work includes a large-scale screening of Arabidopsis accessions to identify those which can recognize AvrRps4^N^. In addition, it would be informative to determine whether AvrRps4^C^ also counteracts the virulence function of AvrRps4^N^ in an *rps4/rrs1* background, or only attenuates the avirulence function of AvrRps4^N^. Our finding that AvrRps4^N^ functions as an effector within plant cells suggests a possible contribution of this moiety when assaying candidate non-bacterial effector molecules by delivery with the Effector Detector Vector 3 (pEDV3), which enables bacterial secretion of effectors into host cells using the first 136 amino acids of AvrRps4 [43]. However, the functions of AvrRps4^N^ encoded in pEDV3 should not interfere with assays using *Pseudomonas* pathogens which contain HopK1 or AvrRps4 natively.

An outstanding question is how AvrRps4^N^ is involved in resistance-triggering in addition to serving as a virulence factor. We previously proposed that EDS1 has roles both upstream and downstream of resistance protein activation [21]. Interactions of AvrRps4^N^ with EDS1 as a guardee may constitute the first step of RPS4/RRS1 activation but is insufficient to trigger immunity in the absence of concurrent RRS1 activation by AvrRps4^C^. Alternatively, AvrRps4^N^ through interactions with EDS1 boosts EDS1 immune signaling in the presence of AvrRps4^C^-activated RPS4/RRS1. Based on the virulence function of AvrRps4^N^ we favor the first model [44]. While additional work will be required to distinguish between these models, our data presented here confirm the interaction between AvrRps4 and EDS1 and clarify the components required for AvrRps4-triggered immunity in Arabidopsis. This is essential for developing an accurate model of RPS4/RRS1 and EDS1 activity in AvrRps4 recognition.

## Materials and methods

### Molecular cloning and plasmid construction

For *in vitro* pull-down assays, GST- EV, AvrRps4^N^, AvrRps4^C^, and AvrRps4^FL^ were cloned into pDEST15 via Gateway recombination as previously described [21]. *EDS1* was cloned into pET28a by inserting amplified *EDS1* into SalI/XhoI using restriction digestion (for all cloning primers, see S1 Table).

Full-length *avrRps4*, *avrRps4*^*N*^, and *avrRps4*^*C*^ were cloned into pVSP_*Ps*SPdes [29] using Gateway recombination. Constructs with the AvrRpm1 signal peptide deleted were made by amplifying the *avrRpm1* promoter with N-terminal EcoRI restriction site and *avrRps4* full-length, *avrRps4*^*N*^, and *avrRps4*^*C*^ with C-terminal HA tags and HindIII sites, and by joining promoter and *avrRps4* fragments with overlap-PCR. The joined clones were then ligated into pVSP_*Ps*SPdes using EcoRI and HindIII. Translational fusions of *avrRps4* full-length, *avrRps4*^*N*^, and *avrRps4*^*C*^ to *GFP* were made by Gateway recombination into the pMDC43 plasmid. Constructs with the AvrRpm1 signal peptide added upstream of AvrRps4 were constructed by digesting AvrRps4-GFP pMDC43 with the PacI enzyme and ligating in the AvrRpm1 signal peptide. pTA7002 vectors were used for generating transgenic Arabidopsis lines expressing Dex-inducible AvrRps4^N^. A single Myc tag was introduced into pTA7002 by amplifying Myc with a 5’ XhoI and 3’ XbaI site. A T4 Polynucleotide Kinase reaction was performed and the insert was ligated into XhoI/SpeI-digested pTA7002. AvrRps4^N^ was cloned into pTA7002-Myc using restriction cloning.

### *In vitro* and *in planta* protein-protein interactions

For *in planta* CoIPs, *Agrobacterium tumefaciens* strains harboring Myc-EDS1 (strain GV3101) and HA-AvrRps4 N-terminus or HA-AvrRps4 C-terminus (strain C58C1) were infiltrated into *N*. *benthamiana* leaves at 0.05 OD. After 48 hours 1 g of tissue was collected and ground in 2 ml buffer H (50 mM HEPES ph7.5, 250 mM sucrose, 15 mM EDTA, 5% glycerol, 3 mM DTT, 0.5% PVPP, 1x Sigma protease inhibitor cocktail). Lysate was centrifuged at 1600 x g for 20 minutes. Supernatant was then spun at 100,000 x g for 1 hour to pellet microsomes. Pellets were resuspended in 1 ml buffer H with 1% NP-40. Protein concentration was measured by Bradford assay, and samples were adjusted to 0.25 mg/ml protein. 20 μl of HA-conjugated beads (Sigma) were added to each sample and incubated at 4°C for 1 hour. Beads were washed three times with buffer H with 0.2% NP-40.

For *in vitro* pull-down assays, BL21(DE3) competent cells were transformed with GST- EV, AvrRps4^N^, AvrRps4^C^, AvrRps4^FL^ or His-T7-EDS1 individually (GST purification) or with His-T7-EDS1 and individual AvrRps4 constructs together (nickel purification). Overnight cultures from fresh colonies were used to inoculate 200 mL LB, incubating 3–4 hours at 37°C under selection. Expression was induced with 0.2 mM IPTG overnight at 22°C. Cultures were centrifuged 10 min at 3800 x g and resuspended in 20 mL TBS with 0.01% NP-40 and 1x cOmplete EDTA-free protease inhibitor (Roche) for GST purification, or equilibration buffer (50 mM sodium phosphate, 300 mM NaCl, 20 mM imidazole, pH 7.4) for nickel purification. Working lysate was prepared by French press lysis followed by centrifugation. Nickel (Fig 1B) or GST (S1B Fig) columns were prepared according to manufacturer’s instructions (Gold Biotechnology and G-Biosciences, respectively, St. Louis, MO, USA). For GST purification, 200 μL bead slurry was washed in each column with 20 mL cold TBS. 10 mL working lysate (AvrRps4) was added to columns and incubated on a rotating shaker for 1 hr at 4°C. Columns were washed with 100x column volume cold TBS. 9.5 ml 2x dilution of HIS-T7-EDS1 lysate was added to each column and incubated shaking at 4°C for 1 hr. Columns were again washed with 100x column volume cold TBS. Protein was eluted from beads by adding 250 μL 10 mM glutathione to the columns and shaking for 15 min at room temperature. For nickel column purification, 2 mL of prepared lysate was passed through 1 mL bead slurry and then washed with 10x column volumes 20 mM imidazole wash buffer, and 1 ml each 50, 100, 150, and 200 mM imidazole wash buffers. Protein was eluted from beads in 250 uL fractions of 250 mM imidazole lysis buffer at 4°C. Immunoblot analysis of input and pulldown fractions were performed using 1:10,000 αGST-HRP and 1:20,000 αT7-HRP (EMD Millipore, Billerica, MA, USA) antibody dilutions.

### Heterologous expression

AvrRps4-GFP-containing plasmids were moved from *E*. *coli* into *Agrobacterium tumefaciens* GV3101 using electroporation. Plants were infiltrated with Agrobacterium at O.D. 0.2. Cells were visualized using a Leica SP8 confocal microscope (Leica Microsystems, Wetzlar, Germany) 3 days post-infiltration. For HR assays in lettuce, N-terminally HA-tagged AvrRps4 full-length, AvrRps4^N^, AvrRps4^C^, HopK1 full-length, HopK1^N^, HopK1^C^, and HA-pBA empty vector were transiently expressed in *Lactuca sativa* cv. *Kordaat* using *A*. *tumefaciens* C58C1 infiltrated at an O.D. of 0.3. Cell death phenotypes were visualized three days post-infiltration. Electrolyte leakage experiments were performed as described previously [45]. Lettuce leaves were infiltrated with agrobacterium strains, and 36 hours later, before HR symptoms become visible, 7 mm leaf discs from 6 individual plants per treatment were harvested, submerged in ddH_2_O, and vacuum-infiltrated. The six leaf disks were then transferred to a 20 mL GC vial containing 10 mL ddH_2_O. Conductivity was measured using a conductivity meter.

### Plant-pathogen interactions

Plants were grown in short-day conditions as previously described [26]. Arabidopsis rosette leaves were infiltrated with bacteria at 5 x 10^4^ cfu/mL densities. Leaf discs were recovered in triplicate samples and thoroughly ground in 10 mM MgCl_2_ and plated on Difco Pseudomonas Agar (Becton, Dickinson and Company, Sparks, MD) containing appropriate antibiotics. Unless noted otherwise, statistical analyses of differential bacterial growth between treatments were performed using ANOVA with Holm correction for multiple comparisons.

### Bacterial *in vitro* secretion and *in planta* cleavage assays

For *in vitro* secretion assays, bacteria were grown on plates containing appropriate antibiotics overnight. Cells were diluted to an O.D. of 2 x 10^8^ cfu/mL into tubes containing 10 mL minimal media [46] at 19°C overnight. Proteins were precipitated using 100% TCA. Total bacterial pellets or precipitated proteins were subjected to immunoblot analysis using HA antibodies (Roche). Total and secreted proteins were subjected to NPTII antibodies to confirm that effectors were not detected in secreted fraction due to cell lysis. For AvrRps4 *in planta* cleavage assays, Col-0 plants were infiltrated with 10^9^ cfu/mL of wild-type DC3000 or DC3000 *hopK1*^*-*^ containing full-length AvrRps4 with or without the AvrRpm1 signal peptide. Tissues were harvested at 0.5 and 7 hpi and ground in 8 M urea for Western blot analysis with HA antibody.

### RT-PCR

Reverse transcription PCR was conducted as described previously [26]. Briefly, total RNA was isolated from indicated plant leaves using TRIzol reagent (Ambion) and RNA was reverse transcribed into cDNA using oligo(dT)15 primers and Moloney murine leukemia virus (MMLV) reverse transcriptase (Promega) according to the manufacturer’s instructions. Equivalent amounts of cDNA were used in each PCR reaction to analyze expression of *avrRps4* and *PR1*. *ACTIN2* was used as an internal control. Oligonucleotide primer sequences used in semi-quantitative RT-PCR are listed in S1 Table.

## Supporting information

S1 Fig AvrRps4^N^ and AvrRps4^C^ interactions with EDS1 are specific. (A) Co-IP of microsomal Myc-EDS1 with GFP-AvrRps4^N^ or AvrRps4^C^-GFP, but not with GFP alone, in *N*. *benthamiana*. For the GFP control, a GFP variant with an ER retention signal was used [47]. (B) *In vitro* interaction of His-T7-EDS1 with GST-AvrRps4^N^ or GST-AvrRps4^C^, but not with GST alone, in *E*. *coli*. These experiments were repeated once with similar results.
(PDF)

S2 Fig Schematic diagrams of AvrRps4 constructs used in this study. (PDF)

S3 Fig *In planta* bacterial growth analysis showing that AvrRpm1SP-AvrRps4 triggers resistance when delivered using the pVSP_*Ps*SPdes system. Col-0 plants were inoculated with 5x10^4^ cfu/mL suspensions of wild-type DC3000 carrying the indicated constructs, and tissue was harvested in triplicate at the 0 and 3 day time points. Values are averages from three independent experiments with triplicate samples, and error bars denote standard deviation, with letters indicating statistically significant differences (*P*<0.0001).
(PDF)

S4 Fig Localization of GFP-tagged AvrRps4 variants in the presence of free RFP. AvrRps4 full-length, AvrRps4^N^, and AvrRps4^C^ with or without the AvrRpm1 signal peptide were C-terminally tagged with GFP and expressed in *N*. *benthamiana* together with free RFP to mark the cytoplasm and nucleus. Cells were visualized after two days.
(PDF)

S5 Fig Resistance to chimeric HopK1^N^-AvrRps4^C^ requires *EDS1* and *RPS4*. *In planta* bacterial growth analysis of DC3000 *hopK1*^*-*^ secreting wild-type AvrRps4 or chimeric HopK1^N^-AvrRps4^C^. Ws-0, *eds1-1* or *rps4-21* plants were inoculated with 5x10^4^ cfu/mL suspensions of bacteria. Values are averages from two independent experiments with triplicate samples, and error bars denote standard deviation, with letters indicating statistically significant differences (*P*<0.01).
(PDF)

S6 Fig Immunoblot analysis confirming secretion of indicated effector proteins. Protein precipitated from the growth medium (left) and total bacterial pellets (right) were subjected to immunoblot analysis using HA (top) antibodies, and NPTII (bottom) antibodies to probe for a non-secreted protein as a control for cell lysis.
(PDF)

S7 Fig *In planta* cleavage assay. Arabidopsis Col-0 leaves infiltrated with 10^9^ cfu/mL of DC3000 or DC3000 *hopK1*^*-*^ containing indicated effectors were harvested at given time points and subjected to immunoblot analysis using HA antibodies. Asterisks indicate full-length or processed C-terminally tagged AvrRps4 proteins, confirming that AvrRpm1SP-AvrRps4 fusion proteins are still processed *in planta*.
(PDF)

S8 Fig *PR1* expression is reduced in the presence of AvrRps4^N^. (A) Transcript levels of *avrRps4* and *PR1* in transgenic plants expressing *avrRps4*^*N*^ (N2 and N8) and wild-type Col-0 by semi-quantitative RT-PCR analysis. Expression of *PR1* was inversely related with that of *avrRps4*^*N*^. Total RNA was isolated from 10-day-old seedlings grown on MS media containing 1 μM Dex (+) or mock (-). *ACTIN2* was used as an internal control. (B) Protein levels of AvrRps4 and PR1 in transgenic plants expressing AvrRps4^N^ (N2 and N8). Total protein was isolated from 3-week-old plants 24 hours after spraying with 50 μM Dex (+) or mock (-). GAPDH was used as a loading control.
(PDF)

S1 Table Primers used in this study. (PDF)
