# Supplementary material for: The bacterial type III-secreted protein AvrRps4 is a bipartite effector
Source: PLoS Pathog. 2018 Mar 30;14(3):e1006984. doi: 10.1371/journal.ppat.1006984 (PMC5895054; doi:10.1371/journal.ppat.1006984)
Supplement: S1 Fig — (A) Co-IP of microsomal Myc-EDS1 with GFP-AvrRps4N or AvrRps4C-GFP, but not with GFP alone, in N. benthamiana. For the GFP control, a GFP variant with an ER retention signal was used [47]. (B) In vitro interaction of His-T7-EDS1 with GST-AvrRps4N or GST-AvrRps4C, but not with GST alone, in E. coli. These experiments were repeated once with similar results. (PDF) [file ppat.1006984.s001.pdf]

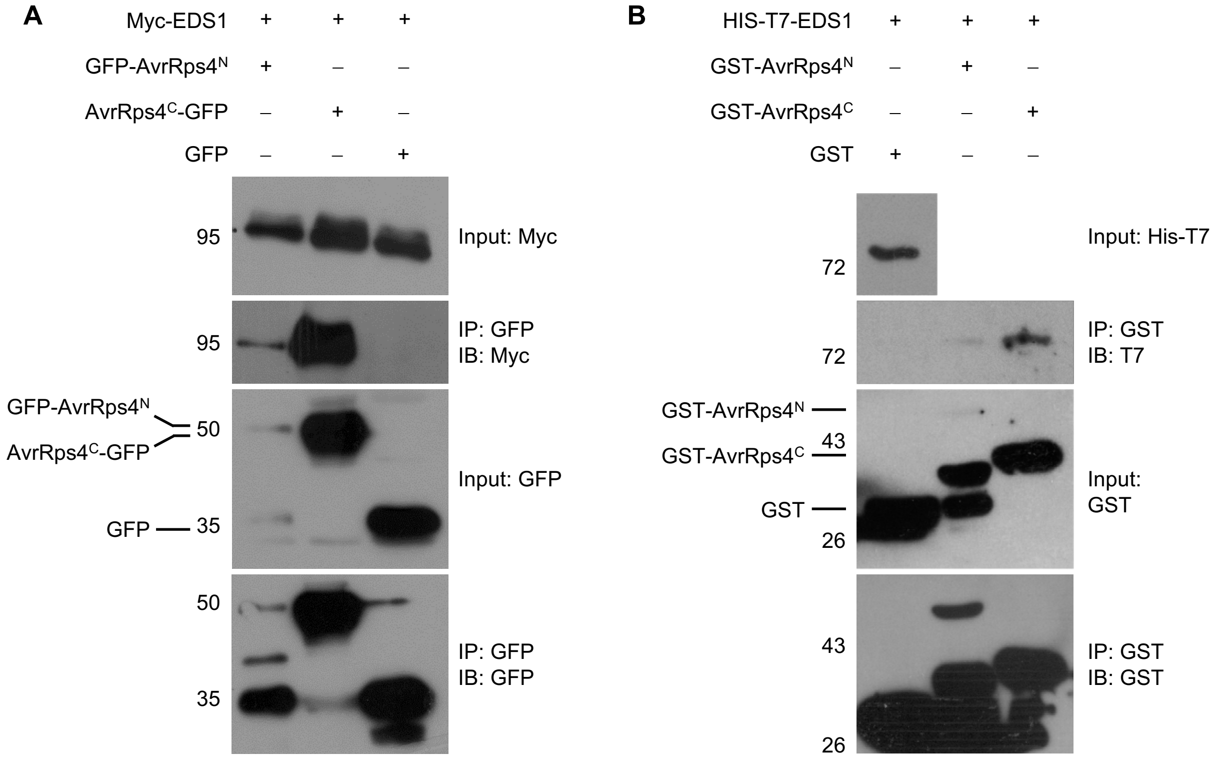

A
Myc-EDS1 + + +
GFP-AvrRps4N + – –
AvrRps4C-GFP – + –
GFP – – +
95
Input: Myc
95
IP: GFP
IB: Myc
GFP-AvrRps4N
AvrRps4C-GFP
50
Input: GFP
GFP
35
50
IP: GFP
IB: GFP
35
B
HIS-T7-EDS1 + + +
GST-AvrRps4N – + –
GST-AvrRps4C – – +
GST + – –
72
Input: His-T7
72
IP: GST
IB: T7
GST-AvrRps4N
43
GST-AvrRps4C
GST
26
Input:
GST
43
IP: GST
IB: GST
26
