# Supplementary figures and images for: The bacterial type III-secreted protein AvrRps4 is a bipartite effector
Source: PLoS Pathog. 2018 Mar 30;14(3):e1006984. doi: 10.1371/journal.ppat.1006984 (PMC5895054; doi:10.1371/journal.ppat.1006984)

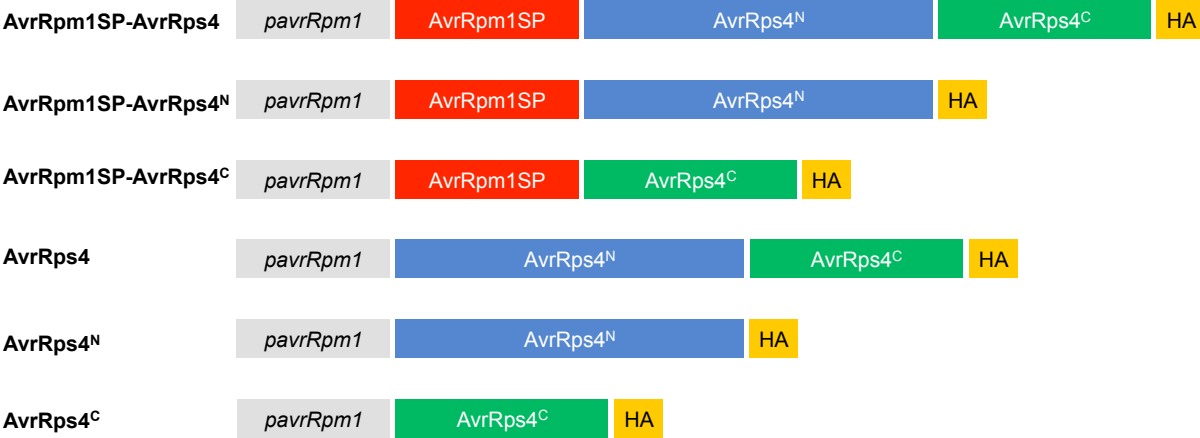

Supplement: S2 Fig — (PDF) [file ppat.1006984.s002.pdf]

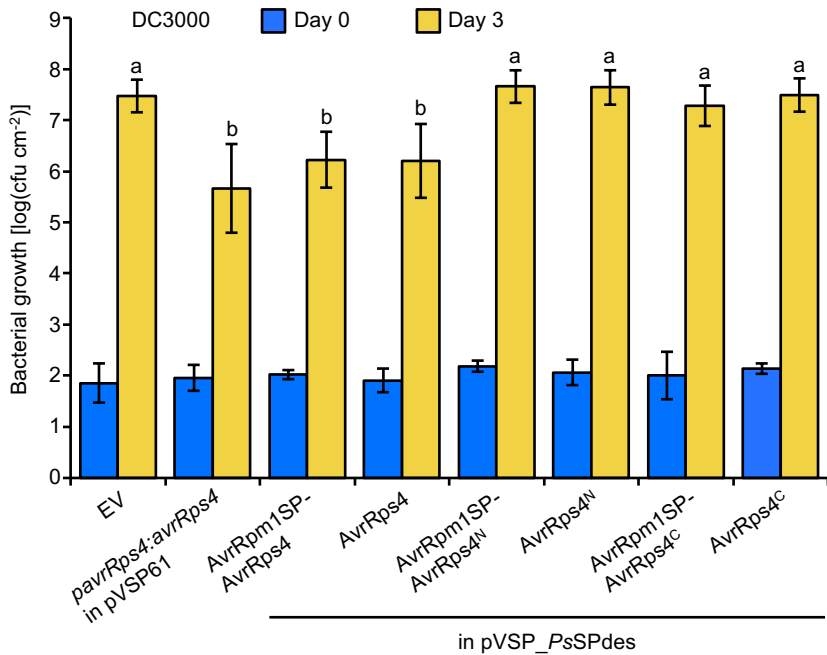

Supplement: S3 Fig — Col-0 plants were inoculated with 5x10^4^ cfu/mL suspensions of wild-type DC3000 carrying the indicated constructs, and tissue was harvested in triplicate at the 0 and 3 day time points. Values are averages from three independent experiments with triplicate samples, and error bars denote standard deviation, with letters indicating statistically significant differences (P<0.0001). (PDF) [file ppat.1006984.s003.pdf]

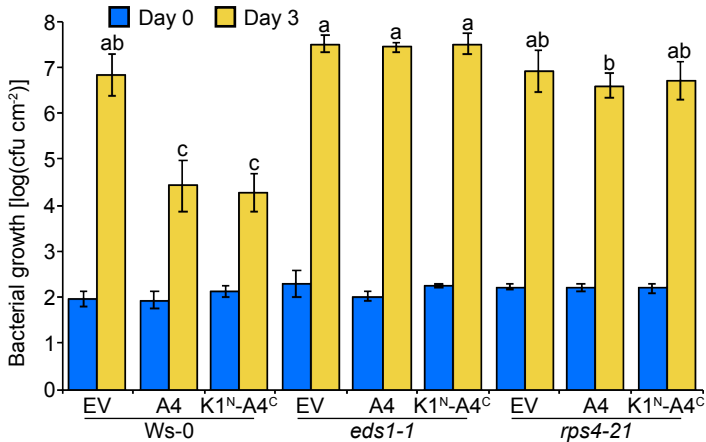

Supplement: S5 Fig — In planta bacterial growth analysis of DC3000 hopK1- secreting wild-type AvrRps4 or chimeric HopK1N-AvrRps4C. Ws-0, eds1-1 or rps4-21 plants were inoculated with 5x10^4^ cfu/mL suspensions of bacteria. Values are averages from two independent experiments with triplicate samples, and error bars denote standard deviation, with letters indicating statistically significant differences (P<0.01). (PDF) [file ppat.1006984.s005.pdf]

**A**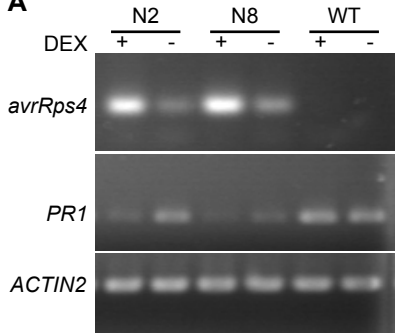**B**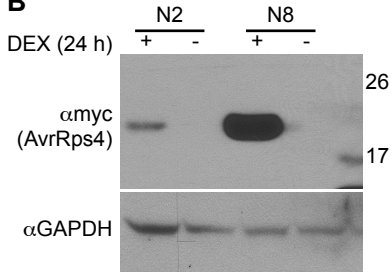

Supplement: S8 Fig — (A) Transcript levels of avrRps4 and PR1 in transgenic plants expressing avrRps4N (N2 and N8) and wild-type Col-0 by semi-quantitative RT-PCR analysis. Expression of PR1 was inversely related with that of avrRps4N. Total RNA was isolated from 10-day-old seedlings grown on MS media containing 1 μM Dex (+) or mock (-). ACTIN2 was used as an internal control. (B) Protein levels of AvrRps4 and PR1 in transgenic plants expressing AvrRps4N (N2 and N8). Total protein was isolated from 3-week-old plants 24 hours after spraying with 50 μM Dex (+) or mock (-). GAPDH was used as a loading control. (PDF) [file ppat.1006984.s008.pdf]
