# Supplementary material for: The bacterial type III-secreted protein AvrRps4 is a bipartite effector
Source: PLoS Pathog. 2018 Mar 30;14(3):e1006984. doi: 10.1371/journal.ppat.1006984 (PMC5895054; doi:10.1371/journal.ppat.1006984)
Supplement: S4 Fig — AvrRps4 full-length, AvrRps4N, and AvrRps4C with or without the AvrRpm1 signal peptide were C-terminally tagged with GFP and expressed in N. benthamiana together with free RFP to mark the cytoplasm and nucleus. Cells were visualized after two days. (PDF) [file ppat.1006984.s004.pdf]

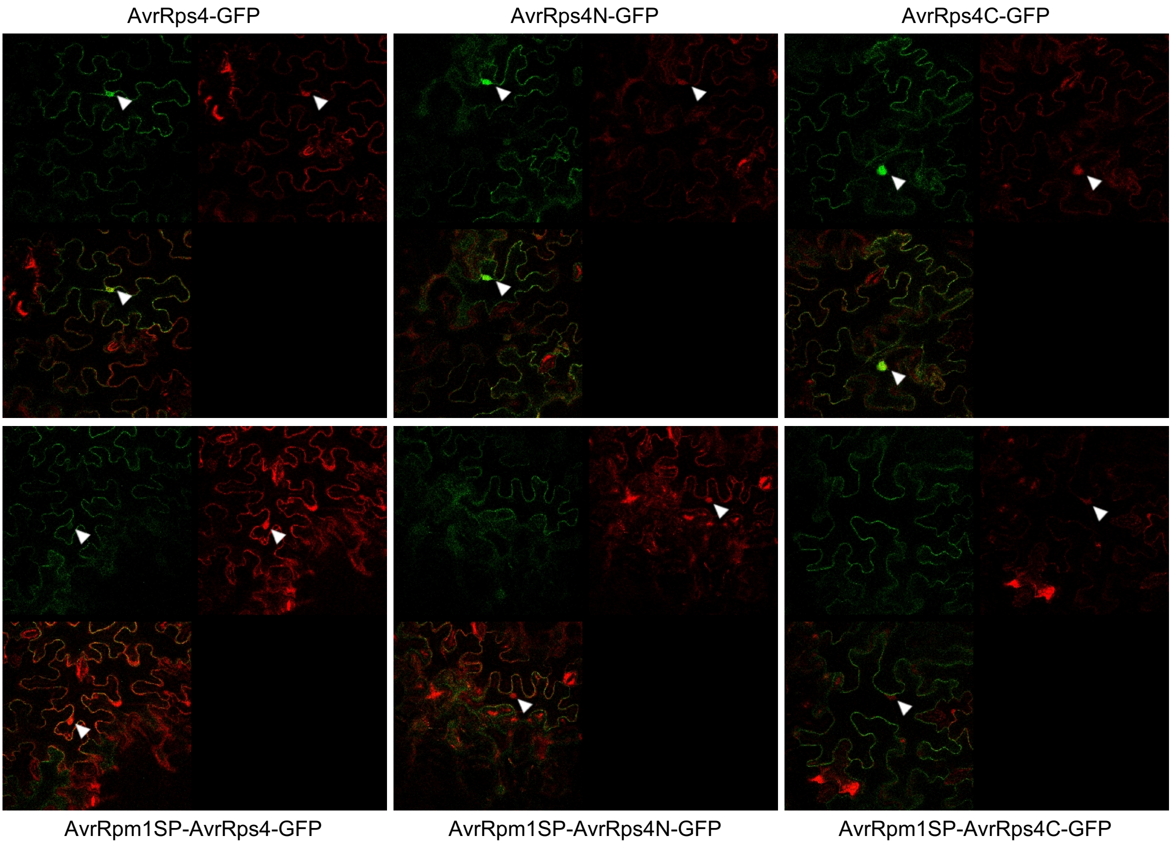
AvrRps4-GFP
AvrRps4N-GFP
AvrRps4C-GFP
AvrRpm1SP-AvrRps4-GFP
AvrRpm1SP-AvrRps4N-GFP
AvrRpm1SP-AvrRps4C-GFP
