# Supplementary material for: The bacterial type III-secreted protein AvrRps4 is a bipartite effector
Source: PLoS Pathog. 2018 Mar 30;14(3):e1006984. doi: 10.1371/journal.ppat.1006984 (PMC5895054; doi:10.1371/journal.ppat.1006984)
Supplement: S6 Fig — Protein precipitated from the growth medium (left) and total bacterial pellets (right) were subjected to immunoblot analysis using HA (top) antibodies, and NPTII (bottom) antibodies to probe for a non-secreted protein as a control for cell lysis. (PDF) [file ppat.1006984.s006.pdf]

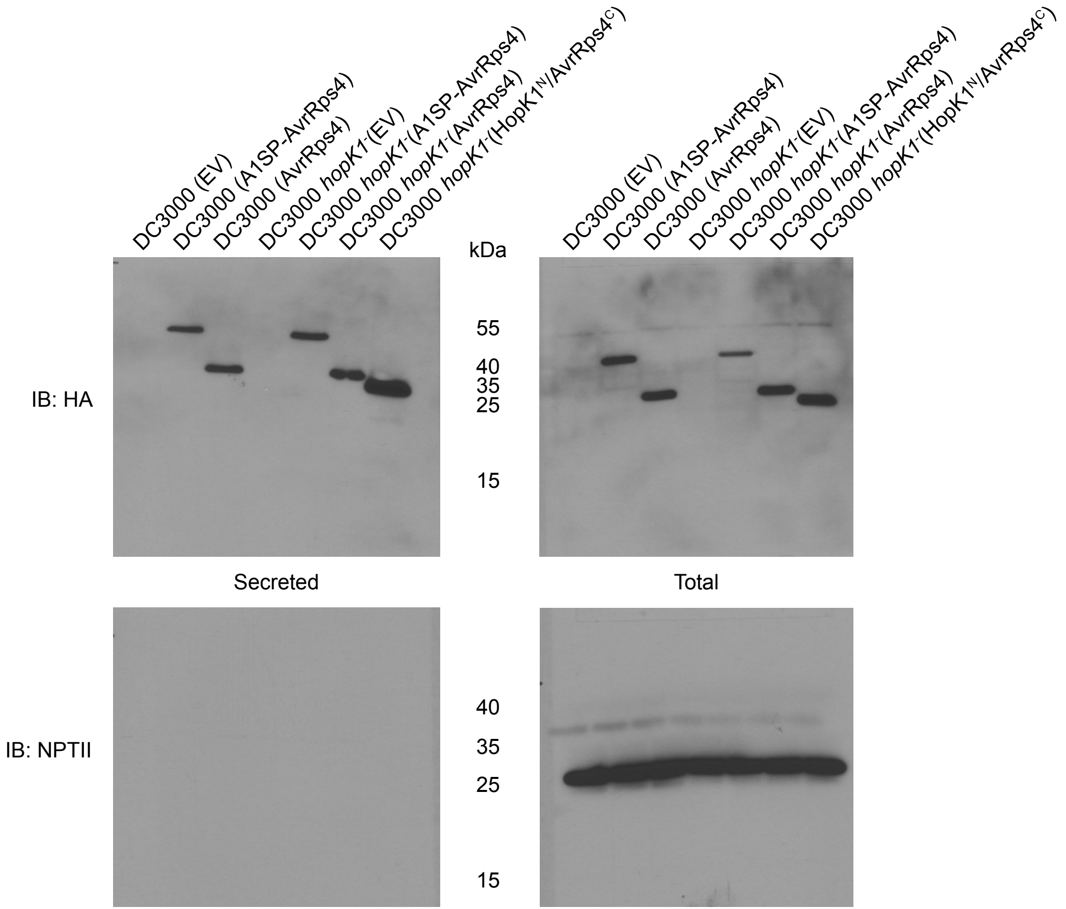
DC3000 (EV)
DC3000 (A1SP-AvrRps4)
DC3000 (AvrRps4)
DC3000 hopK1⁻(EV)
DC3000 hopK1⁻(A1SP-AvrRps4)
DC3000 hopK1⁻(AvrRps4)
DC3000 hopK1⁻(HopK1N/AvrRps4C)
kDa
55
40
35
25
15
IB: HA
Secreted
Total
IB: NPTII
40
35
25
15
