# Supplementary material for: The bacterial type III-secreted protein AvrRps4 is a bipartite effector
Source: PLoS Pathog. 2018 Mar 30;14(3):e1006984. doi: 10.1371/journal.ppat.1006984 (PMC5895054; doi:10.1371/journal.ppat.1006984)
Supplement: S7 Fig — Arabidopsis Col-0 leaves infiltrated with 109 cfu/mL of DC3000 or DC3000 hopK1- containing indicated effectors were harvested at given time points and subjected to immunoblot analysis using HA antibodies. Asterisks indicate full-length or processed C-terminally tagged AvrRps4 proteins, confirming that AvrRpm1SP-AvrRps4 fusion proteins are still processed in planta. (PDF) [file ppat.1006984.s007.pdf]

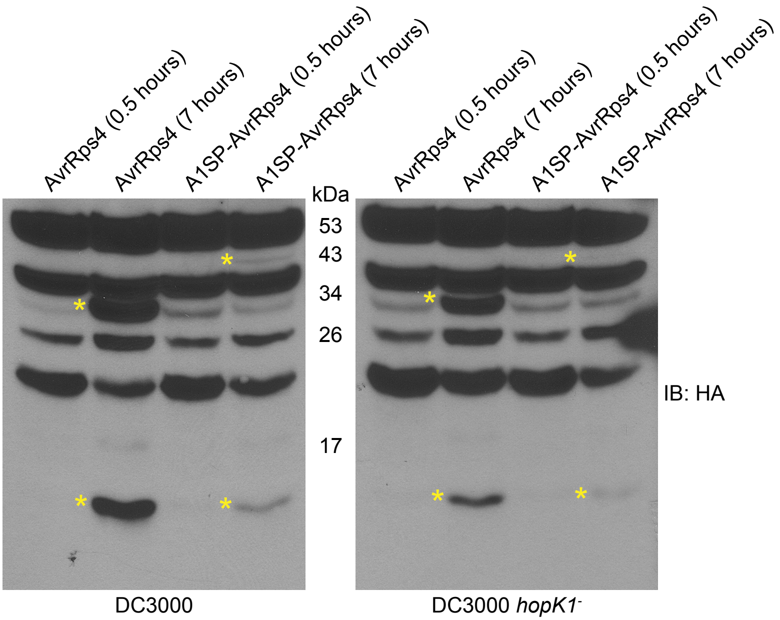
AvrRps4 (0.5 hours)
AvrRps4 (7 hours)
A1SP-AvrRps4 (0.5 hours)
A1SP-AvrRps4 (7 hours)
AvrRps4 (0.5 hours)
AvrRps4 (7 hours)
A1SP-AvrRps4 (0.5 hours)
A1SP-AvrRps4 (7 hours)
kDa
53
43
34
26
17
IB: HA
DC3000
DC3000 *hopK1*-
