# Supplementary material for: The bacterial type III-secreted protein AvrRps4 is a bipartite effector
Source: PLoS Pathog. 2018 Mar 30;14(3):e1006984. doi: 10.1371/journal.ppat.1006984 (PMC5895054; doi:10.1371/journal.ppat.1006984)
Supplement: S1 Table — (PDF) [file ppat.1006984.s009.pdf]

Supplemental Table 1. Primers used in this study

Primers	Sequence 5'-3'
attB1 AvrRps4 FOR	AAAAAGCAGGCTCAATGAATCGAATTTCAACC
attB1 AvrRps4 REV	AGAAAGCTGGGTATTGGTTGATTCTGCGGTCT
attB1 AvrRps4C FOR	AAAAAGCAGGCTCAATGGGTAAACGAGTCTATCAAATT
attB1 AvrRps4N REV	AGAAAGCTGGGTATCCGCCCAATAGGGATTTGG
AvrRpm1 Promoter FOR	GGGGGAATTCGGAGGCCTGCAGAATTCGGCA
HA REV	GGGGAAGCTTTCATGCGTAGTCTGGTACG
SpeI AvrRps4 FOR	GGACTAGTATGACTCGAATTTCA
SpeI AvrRps4 REV	GGACTAGTTTATTGGTTGATTCT
KpnI AvrRps4 FOR	GGGGTACCATGACTCGAATTTCA
KpnI AvrRps4 REV	GGGGTACCTTGGTTGATTCTGCG
EDS1 Sal1 FOR	TTTTGTCGACAAATGGCGTTTGAAGCTC
EDS1 XhoI REV	AAAACTCGAGTCAGGTATCTGTTATTT
StgXhoI-XbaI-Myc	TCGAGCCCGGGACTAGTATGGAACAAAAACTTATTTCTGAAGAAG ATCTGTGAT
ComStgXhoI-XbaI-Myc	CTAGATCACAGATCTTCTTCAGAAATAAGTTTTTGTTCCATACTAGT CCCGGGC
XhoI AvrRps4 FOR	GGCTCGAGATGACTCGAATTTCAACC
SpeI AvrRps4 REV	GGACTAGTTTGGTTGATTCTGCGGTC
SpeI AvrRps4N Rev	GGACTAGTTCCACCCAATAGGGATTT
XhoI AvrRps4C FOR	GGCTCGAGATGGGTAAACGAGTCTATCA
PR1 FOR	GCAATGGAGTTTGTGGTCAC
PR1 REV	GTTCACATAATTCCCACGAGG
ACTIN2 FOR	TCGGTGGTTCCATTCTTGCT
ACTIN2 REV	GCTTTTTAAGCCTTTGATCTTGAGAG
AvrRps4 FOR	ATGACTCGAATTTCAACC
AvrRps4N REV	GGTCCACCCAATAGGGATTTGGGTG
